# Preventive practices and parental attitudes towards snakebites in children in snakebite hotspots of rural Sri Lanka

**DOI:** 10.1136/bmjpo-2025-003543

**Published:** 2025-06-13

**Authors:** Kavinda Dayasiri, Gihan Gunarathna, Indika Gawarammana, Shaluka Jayamanne

**Affiliations:** 1 University of Kelaniya, Kelaniya, Sri Lanka; 2 University of Peradeniya, Peradeniya, Sri Lanka

**Keywords:** Toxicology, Child Health, Epidemiology

## Abstract

**Background:**

Snakebites remain a major public health issue in rural Sri Lanka, particularly among children under 5. Parental attitudes, knowledge and preventive practices significantly influence the risk of snakebites and the effectiveness of first aid responses. This study aimed to assess parental attitudes, knowledge sources and preventive practices related to snakebite prevention and management in children living in snakebite-endemic rural regions of Sri Lanka.

**Methods:**

A descriptive cross-sectional study was conducted in the Ampara and Polonnaruwa Districts, two snakebite-endemic regions in Sri Lanka. The study targeted parents with at least one child under 5 years old, who were selected through cluster sampling facilitated by Public Health Midwives. A structured, self-administered questionnaire was used to collect data on demographic characteristics, parental attitudes towards snakes and snakebites, knowledge sources and preventive practices.

**Results:**

A total of 518 parents participated, with the majority being mothers (94.2%). Extreme fear of snakes was reported by 92.7% of participants. Parental beliefs varied, with 23.7% believing that a snake should always be killed after biting a person and 18.0% holding the belief that snakes take revenge. Significant associations were found between extreme fear of snakes and the absence of prior training in snakebite first aid (p=0.035) as well as being a mother (p=0.001). Major challenges in snakebite care included transportation difficulties (90.5%), lack of proximity to hospitals with emergency treatment (81.5%) and reliance on traditional healing practices (32.6%). Traditional healing practices (32.6%) were significantly associated with low socioeconomic status (p=0.001) and low parental education (p=0.001). Social media (42.1%) was the most common source of knowledge on snakebite care. Storing paddy at home was significantly associated with a family history of snakebites (p=0.004)

**Conclusions:**

Parental fear, cultural beliefs and limited access to training programmes significantly influence snakebite prevention and management practices. Enhancing community-based education, improving healthcare accessibility and incorporating snakebite first-aid training into public health programmes could mitigate the risks and improve outcomes for paediatric snakebite cases in rural Sri Lanka.

**WHAT IS ALREADY KNOWN ON THIS TOPIC**

- Snakebites are a significant public health concern in rural Sri Lanka, with children being particularly vulnerable.
- Parental attitudes, knowledge and preventive practices play a crucial role in mitigating snakebite risks.
- Limited awareness and reliance on traditional beliefs can influence healthcare-seeking behaviour and first-aid responses.

**WHAT THIS STUDY ADDS**

- Identifies key determinants of parental attitudes towards snakes and snakebites, including fear, first-aid training and socioeconomic factors.
- Highlights gaps in parental knowledge and preventive practices.
- Demonstrates that social media is the primary source of snakebite-related information for parents.

**HOW THIS STUDY MIGHT AFFECT RESEARCH, PRACTICE OR POLICY**

- Emphasises the need for targeted educational programmes to improve snakebite-related attitudes among parents.
- Supports the development of community-based interventions to address misconceptions and enhance preventive measures.
- Provides evidence for policy-makers to integrate structured snakebite education into maternal and child healthcare initiatives.

## Introduction

 Snakebites are a significant but often neglected public health problem, contributing to substantial morbidity and mortality globally, especially in tropical and subtropical regions. According to the WHO, approximately 5.4 million snakebites occur each year, leading to up to 138 000 deaths, with a disproportionate burden falling on rural and economically disadvantaged communities.^1^ Children are particularly vulnerable due to their smaller body size and behaviours that increase exposure to snakes.^2^

In South Asia, countries such as India, Bangladesh, Nepal and Sri Lanka experience a high incidence of snakebites, particularly in rural agricultural settings.^3^ Socioeconomic factors, cultural beliefs and limited access to healthcare significantly affect the outcomes of snakebite incidents. Studies from India and Bangladesh have shown that preventive practices, such as wearing protective footwear and using bed nets, remain inconsistently practised, and that misconceptions about snakes and reliance on traditional healers persist.^4 5^

In Sri Lanka, snakebites are a recognised public health issue, particularly in rural districts.^6^ While several studies have described the epidemiology and clinical management of snakebites in adults, there is limited research focusing specifically on preventive practices and parental attitudes towards snakebites in children.^7^ In snakebite-endemic districts such as Ampara and Polonnaruwa, children’s exposure to agricultural and outdoor environments further elevates their risk, yet little is known about how parental behaviours and knowledge influence snakebite prevention and management. This study, therefore, aimed to explore parental attitudes, knowledge sources and preventive actions related to snakebites in children, focusing on the challenges that hinder effective care and preventive practices in rural communities.

## Methods

### Study design and population

A descriptive cross-sectional study was conducted in the Ampara and Polonnaruwa Districts of Sri Lanka, both of which are rural areas with a high incidence of snakebites.^8^ The target population consisted of parents with at least one child under 5 years of age, who were permanent residents in these districts. Public Health Midwives (PHMs) from the area facilitated participant identification based on birth and immunisation records maintained at the local Medical Officer of Health offices.

### Sample size and sampling procedure

To determine the appropriate sample size, the study referenced the work of Silva *et al*^8^ and applied the standard formula for a descriptive cross-sectional study. With a 95% confidence level (Z=1.96), an estimated proportion (p=0.76), and a 5% margin of error (d=0.05), the sample size was calculated to be 281 participants. Cluster sampling was used, with each PHM region representing a cluster. The study involved 197 PHM regions (94 from Polonnaruwa and 103 from Ampara). Within each region, 2–3 eligible parents were randomly selected. The inclusion criteria were parents who were permanent residents of Ampara or Polonnaruwa, had at least one child under 5 and provided informed consent. Exclusion criteria included parents who reported having significant cognitive impairments or mental health conditions that might interfere with their ability to accurately recall and report knowledge and practices related to snakebite prevention and management. Parents who were unable to provide informed consent were also excluded. Only permanent residents of the selected districts were included in the study to ensure participants had consistent access to local healthcare services and public health initiatives. Temporary residents, who might not have had the same exposure to the local healthcare system, health programmes or community-specific risk factors, were excluded to maintain the validity of the data.

### Data collection

Data collection was carried out for 6 months (from October 2024 to March 2025) using structured, self-administered questionnaires provided to the parents by the respective PHMs. The questionnaires, administered in Sinhala or Tamil based on the participants’ preference, were divided into three sections: Demographic details (age, gender, education, ethnicity, socioeconomic status, prior training in first aid for snakebites), parental attitudes towards snakes and snakebites, sources of knowledge and preventive practices by parents for snakebites in their children.

The reliability of the questionnaire was ensured through a pretest with 10 parents from non-study areas to identify any issues with clarity and consistency. Validity was established through expert review for content validity and pretesting with participants for face validity. Feedback from these processes was used to refine the questionnaire for clarity and relevance.

### Study variables and definitions

The study examined several key variables related to challenges in providing care for children with snakebites. Monthly household income was considered as the total income of all earners in the family during a month, as reported by the participant. Transportation difficulties were defined as parents’ subjective perception of limited or unreliable means of transport that delay or prevent timely access to healthcare facilities. Lack of proximity to hospitals with emergency treatment units was defined as the absence of a healthcare facility within 20 km from home that is equipped to provide emergency snakebite management, including antivenom administration. Traditional beliefs and customs among parents included cultural or community-driven practices, such as reliance on traditional healers or non-medical treatments, that influenced healthcare-seeking behaviour. Lack of exposure to effective training programmes was defined as the absence of structured educational opportunities for caregivers on prehospital management of snakebites. Deficiencies in existing community-based training programmes referred to parents’ perception of inadequacies in initiatives aimed at improving public awareness and preparedness for snakebite prevention and management. Lack of financial resources was defined as parents’ subjective perception of economic constraints limiting access to transportation, healthcare services or necessary medical interventions. Low socioeconomic status was categorised as a monthly household income of US$600 or less. Parents were considered to have prior personal experience with snakebites if they had previously cared for someone who had suffered a snakebite. Low parental education was defined as not having completed grade 10. Parents who had undergone any form of first-aid training specifically related to managing snakebites in children—whether through healthcare providers or community-based programmes—were identified as having prior first-aid training for paediatric snakebites.

### Data analysis

Data were analysed using SPSS software V.26.0. Normality of continuous variables (such as age and household income) was assessed using the Shapiro-Wilk test and by visual inspection of histograms and Q-Q plots. Continuous variables with a normal distribution were summarised using mean and SD, while non-normally distributed variables were summarised using median and IQR. Descriptive statistics, including frequency distributions, were used to summarise categorical data. χ^2^ tests were applied to assess associations between demographic factors and parental attitudes and preventive practices. Variables that demonstrated a p<0.1 in bivariate analyses were included in a multivariable logistic regression model to identify independent factors associated with parental attitudes and preventive practices. Adjusted ORs (AORs) with 95% CIs were reported. A p<0.05 was considered statistically significant.

### Patient and public involvement

Patients and/or the public were not involved in the design, or conduct, or reporting, or dissemination plans of this research.

## Results

A total of 518 parents from the Ampara and Polonnaruwa Districts participated in the study. The normality of continuous variables was assessed using the Shapiro-Wilk test and visual inspection of histograms and Q-Q plots. Parental age was found to be normally distributed (Shapiro-Wilk p=0.124, mean 27.0 years (SD=6.4). Monthly household income, however, was non-normally distributed (Shapiro-Wilk p<0.001, median US$265, IQR: US$225–US$295).

The ethnic composition of the sample included 87.6% (n=454) Sinhalese, 7.9% (n=41) Muslim and 4.4% (n=23) Tamil parents. Prior snakebite exposure was rare, with only 4 parents (0.4%) reporting that their child had experienced a snakebite, while 13.7% (n=71) had a family member who had been bitten by a snake in the past. Only 30 (5.8%) of parents reported receiving training on the correct method of immobilisation on a child with a snakebite, while 488 (94.2%) had not.

### Parental attitudes towards snakes and snakebites

The majority of participants (480; 92.7%) reported being extremely afraid of snakes. Moreover, 123 (23.7%) believed that a snake should always be killed after biting a person, while 395 (76.3%) disagreed with this notion. A smaller proportion (93; 18.0%) held the belief that a snake remembers and takes revenge on those who tease it, whereas 425 (82.0%) did not support this idea. Additionally, 298 (57.5%) of respondents considered the cobra to be a sacred animal, while 220 (42.5%) did not share this belief. Only 6 out of 518 parents (1.2%) had previously provided first aid to a child with a snakebite, while 29 parents (5.6%) had received prior training in administering first aid for paediatric snakebites. The effect of selected determinants of parental attitudes towards snakes and snakebites is shown in table 1.

**Table 1:** Determinants of parental attitudes towards snakes and snakebites

Attitude	No training in first-aid		Experience in first-aid		Mother parent		Family history of snakebites	
	χ^2^	P value	χ^2^	P value	χ^2^	P value	χ^2^	P value
Extreme fear of snakes	4.43	0.03	0.77	0.37	11.98	0.001	0.15	0.698
Snake should always be killed after a bite	3.04	0.08	1.89	0.17	3.32	0.07	0.31	0.57
An unharmed snake will seek revenge later	6.72	0.01	0.01	0.93	5.11	0.02	2.49	0.12
Cobra is a sacred animal	0.42	0.52	0.14	0.70	2.62	0.11	9.37	0.002

### Determinants of parental attitudes towards snakes and snakebites

Determinants of extreme fear of snakes included no prior training in snakebite first aid (p=0.035) and being a mother (p=0.001). Family history of snakebites was not significantly associated with extreme fear (p=0.698). None of the variables were associated with parents’ determination to kill the snake but was associated with extreme fear (χ^2^=4.98, p=0.03). The belief that a snake takes revenge was significantly associated with no prior training in snakebite first aid (p=0.010) and being a mother (p=0.024). No significant association was found with experience in first aid (p=0.934) or family history of snakebites (p=0.114). The belief that the cobra is a sacred animal was not significantly associated with first aid involvement, training or being a mother. However, a significant difference was observed in family history of snakebites, with a higher proportion of those who did not consider the cobra sacred having a family member bitten (p=0.002). The challenges perceived by parents in providing care for children with snakebites are presented in table 2.

**Table 2:** Challenges to giving care for children with snakebites

Challenges to giving care for children with snakebites	Yes	No	Low socioeconomic status		Low parental education	
			χ^2^	P value	χ^2^	P value
Transportation difficulties	469 (90.5%)	49 (9.5%)	7.68	0.002	1.92	0.068
Lack of proximity to hospitals with emergency treatment units	422 (81.5%)	96 (18.5%)	13.46	0.001	2.98	0.045
Traditional beliefs and customs among parents	349 (32.6%)	169 (67.4%)	10.82	0.001	12.32	0.001
Lack of exposure to effective training programmes	471 (90.9%)	47 (9.1%)	3.22	0.042	9.83	0.001
Deficiencies in existing community-based training programmes	309 (59.7%)	209 (40.3%)	1.05	0.129	1.71	0.082
Lack of financial resources	363 (70.1%)	155 (29.9%)	14.42	0.001	3.45	0.034

Several challenges impacted care for children with snakebites. Transportation difficulties (90.5%) and lack of proximity to emergency hospitals (81.5%) were significantly associated with low socioeconomic status. Traditional beliefs (32.6%) and lack of training exposure (90.9%) were linked to both low socioeconomic status and low parental education. Financial constraints (70.1%) also showed significant associations with these factors. However, deficiencies in community-based training programmes (59.7%) did not have significant associations.

### Multivariable regression analysis

Logistic regression analysis identified that lack of prior training in first aid (AOR 2.18, 95% CI 1.12 to 4.26, p=0.022) and being a mother (AOR 3.07, 95% CI 1.45 to 6.51, p=0.003) were independent predictors of extreme fear of snakes. Similarly, lower household income (AOR 2.59, 95% CI 1.32 to 5.08, p=0.006) and lower parental education level (AOR 2.17, 95% CI 1.11 to 4.24, p=0.023) were independent predictors of reliance on traditional healing practices.

### Sources of knowledge on snakebite care among parents

The main sources of knowledge regarding snakebite care among parents were social media (42.1%), followed by personal experience (18.3%) and education or training programmes (14.5%). Other sources included advice from colleagues (9.7%), television (7.7%), printed media (5.4%) and radio (2.3%).

### Method of knowledge search by parents following a snakebite

In response to how parents search for knowledge following a snakebite, the majority, 83.2% (431), indicated they would call a doctor for guidance. A smaller portion, 7.1% (37), would contact the poison information centre, while 3.9% (20) would refer to a national guideline. Additionally, 3.5% (18) would turn to online sources for information, and 2.3% (12) would call a friend for advice.

### Prevention practices

Regarding preventive measures, most participants reported that their children do not sleep on the floor at night, with only 2 (0.4%) indicating that their child does sleep on the floor. A significant majority, 492 (95%), stated that their child sleeps under a mosquito net, while only 26 (5%) did not. In terms of personal safety, 191 (36.9%) of participants wore safety shoes or boots when walking in the dark, whereas 327 (73.1%) did not. The vast majority, 511 (98.6%), reported using a flashlight or light source when walking in the dark, with only 7 (1.4%) not using any light source. Regarding household factors, 138 (26.6%) participants had a rice store at their house, while 280 (73.4%) did not. Additionally, 387 (74.7%) participants reported having ant holes in their garden, with 131 (25.3%) not having any.

Among the risk factors examined, storing rice at home was the only significant factor (p=0.004) associated with a family history of snakebite (table 3). Other risk factors, including whether the child sleeps on the floor at night (p=0.572), the presence of ant hills in the home garden (p=0.145), wearing safety shoes or boots when walking in the dark (p=0.205), using a flashlight or light source when walking in the dark (p=0.25), and whether the child sleeps under a mosquito net (p=0.799), did not show significant associations.

**Table 3:** Association between risk factors and family history of snakebite among participants

Risk factor	Family history of a snakebite		χ^2^	P value
	Yes (n=71)	No (n=447)		
Child sleeps on the floor at night	0 (0%)	2 (0.4%)	0.319	0.572
Ant hills in the home garden	58 (81.6%)	329 (73.6%)	2.121	0.145
Store rice at the house	29 (40.8%)	109 (24.3%)	8.494	0.004
Wear safety shoes/boots when walking in the dark	21 (29.5%)	167 (37.3%)	1.605	0.205
Use a flashlight/light source when walking in the dark	69 (97.1%)	442 (98.8%)	1.326	0.254
Child sleeps under a mosquito net	67 (94.3%)	425 (95.0%)	0.065	0.799

## Discussion

This study highlights several critical findings regarding parental attitudes, knowledge sources and preventive practices related to snakebites among families in rural Sri Lanka. A majority of parents (92.7%) reported extreme fear of snakes, and significant gaps were noted in first-aid knowledge, with only 5.8% having received prior training. These findings are consistent with previous research conducted in rural South Asian regions, where cultural beliefs, fear and limited formal education about snakebite management are common.^9^ Culturally sensitive educational interventions and community awareness programmes have been shown to reduce fear and improve appropriate responses to snake encounters, ultimately aiding in better snakebite prevention and management.^10 11^ Despite these general safety measures, more specific preventive actions, such as wearing safety shoes when walking at night, were less common (36.9%). This finding is consistent with other studies from rural areas, where, while basic protective actions are practiced, more proactive measures tend to be less emphasised.^12^

In terms of knowledge, social media emerged as the dominant source (42.1%), followed by personal experiences (18.3%) and education or training programmes (14.5%). These results are comparable to other studies conducted in rural settings, where information via digital platforms has surpassed traditional means such as television and printed media as the primary source of health-related knowledge.^13^ Studies from other resource-limited settings highlighted the role of social media in disseminating health information, including snakebite prevention, particularly in remote communities.^14^ However, relying on social media without sufficient guidance may lead to misinformation, which may be detrimental in crisis situations like snakebites.^15^

Traditional beliefs and customs were found to be prevalent in the rural Sri Lankan context, as evidenced by the finding that 23.7% of parents believed a snake should always be killed after a bite. Additionally, 18% of participants held the belief that snakes seek revenge after being harmed. Similarly, studies in other resource-limited countries in South Asia and Africa have reported the persistence of traditional beliefs influencing snakebite treatment, including reliance on traditional healers instead of seeking medical attention.^4 16 17^ These practices, while culturally ingrained, pose significant challenges to effective management and prevention of snakebites. Socioeconomic factors were found to significantly influence parental practices and attitudes towards snakebites. Challenges such as transportation difficulties (90.5%) and lack of proximity to hospitals with emergency treatment units (81.5%) were reported as major barriers to effective snakebite management. These findings are consistent with studies from other snakebite-endemic regions, such as rural India, Nepal and Brazil, where access to healthcare is often limited due to long distances, poor infrastructure and financial constraints.^5 17 18^ A study from Nepal further highlighted that the financial burden of snakebite care can exacerbate access barriers, with significant impacts on household economies and care-seeking behaviour.^19^

The authors acknowledge the limitations of this study, including its cross-sectional design, limited generalisability to other regions and reliance on self-reported data, which may have introduced recall bias. Future studies should address these limitations by employing longitudinal designs, expanding the sample size to include diverse populations and using objective measures of snakebite incidence and treatment outcomes to refine the understanding of parental knowledge and practices.

## Conclusions

This study highlights significant gaps in parental knowledge and training regarding snakebite management, with limited prior first aid training and a high prevalence of extreme fear of snakes, especially among mothers. Socioeconomic factors, such as lower household income and education levels, were associated with reliance on traditional healing practices and challenges in providing care. The findings underscore the need for culturally sensitive targeted educational programmes to improve awareness and first aid skills among parents living in snakebite-endemic rural regions of Sri Lanka.

## Data Availability

Data are available on reasonable request.
