# Supplementary material for: Preventive practices and parental attitudes towards snakebites in children in snakebite hotspots of rural Sri Lanka
Source: BMJ Paediatr Open. 2025 Jun 13;9(1):e003543. doi: 10.1136/bmjpo-2025-003543 (PMC12164630; doi:10.1136/bmjpo-2025-003543)
Supplement: Supplementary file 1 [file bmjpo-2025-003543.reviewer_comments.pdf]

PEER REVIEW HISTORY

BMJ Paediatrics Open publishes all reviews undertaken for accepted manuscripts. Reviewers are asked to complete a checklist review form and are provided with free text boxes to elaborate on their assessment. These free text comments are reproduced below.

ARTICLE DETAILS

**TITLE (PROVISIONAL)**	Preventive practices and parental attitudes towards snakebites in children in snakebite hotspots of rural Sri Lanka
**AUTHORS**	Dayasiri, Kavinda; Gunarathna, Gihan; Gawarammana, Indika; Jayamanne, Shaluka

VERSION 1 - REVIEW

**REVIEWER NAME**	***Deb Pandey***
**REVIEWER AFFILIATION**	Agriculture and Forestry University, Department of Veterinary Microbiology and Parasitology
**REVIEWER CONFLICT OF INTEREST**	N/A
**AI DISCLOSURE**	No
**DATE REVIEW RETURNED**	19-Apr-2025

**GENERAL COMMENTS**	bmjpo-2025-003543_Proof_hi<br><br>General<br>Authors have nicely presented about their attitudes, knowledge, and behaviours of mothers of 5-year-old children towards snakes and snakebite in two rural districts of Sri Lanka (one of the snakebite hotspots in South Asia). However, they need to revise evaluation of household income and data analysis, etc. in method section and first paragraph of the discussion section prior to its accepting for publication. I provided some comments specifically below.<br><br>Authors have presented background of this study nicely. But, authors need to define the constraint of this study at the last of discussion that would help to refine the similar studies in future.<br><br>Specific<br>Line 54, 33-34: Please, replace "Ampara and Polonnaruwa districts" with Ampara and Polonnaruwa Districts. It is because common names are capitalized.<br>Please, place US Dollar equivalent amount in the parenthesis of 200,000 LKR. Next, please, elaborate the monthly household income for farmers who receive cash from majority of crops bi-, tri- or four monthly or so on. The rural areas of this study sites would be dominated by farmers. How did you measured or defined monthly income? Please, elaborate this section, herein.<br><br>Page 6, line 3: means<br>The mean is improper descriptive statistics if the data were non-normally distributed. So, please, state about the normality of your dataset for each of these variables being analyzed and use either mean or median according to the results of normality tests. Otherwise, the method is relatively weak. To improve the method section and finding robust findings, too, I would like to suggest

	author to apply regression analyses to find and declare the most strong factors affecting attitudes and practices of the parents.<br><br>Page 10, 13-20: The first paragraph of the discussion is to highlight your core findings and support or argue with other similar findings from other rural areas of Sri Lanka or other South Asian countries where socio-economics and cultural practices are similar.<br>Please, remove "Snakebites in rural Sri Lanka, particularly in districts like Ampara and Polonnaruwa, continue to be a significant public health issue8. These regions, identified as snakebite hotspots due to their vast agricultural lands and favorable habitats for snake populations, witness a high incidence of envenomations annually2. Among the most vulnerable groups are children, whose curiosity and mobility increase their risk of encountering snakes9." From the first section of this paragraph.

**REVIEWER NAME**	***Godpower Michael***
**REVIEWER AFFILIATION**	Aminu Kano Teaching Hospital, Department of Family Medicine
**REVIEWER CONFLICT OF INTEREST**	N/A
**AI DISCLOSURE**	No
**DATE REVIEW RETURNED**	26-Apr-2025

**GENERAL COMMENTS**	GENERAL COMMENTS<br>Snakebite remains a neglected tropical disease. The topic of this study, “Preventive practices and parental attitudes towards snakebite in children in snakebite hotspots of rural Sri Lanka,” is an important contribution to the Sri Lankan literature. The grammatical style of the authors is easy to understand, and the technical presentation of the tables is commendable. The discussion and conclusions are okay. However, there are a few issues in the Introduction, methods, and results sections that need to be addressed.<br>SPECIFIC COMMENTS<br>Abstract<br>1. The aim of the study was not clearly stated.<br>Introduction<br>2. Here, I struggled to find current evidence on preventive practices and parental attitudes towards snakebite in children. Using the funnel approach, the reader would be interested in seeing a concise description of what is known of this topic at the global, regional, national, and local levels. This helps expose the gap in the current knowledge, which this study is designed to address.<br>Methods<br>3. Lines 29-33: It appears that the aim of the study is inconsistent with the one stated in lines 19-25. Please, the aim of the study should be consistently stated for clarity.<br>4. When was this study carried out?<br>5. Page 4, Line 47: Do you mean that p=43 % was obtained from the paper written by Silva et al? If so, which variable does this represent (parental attitude or preventive practices)? Transparency is required in calculating the sample size.<br>6. In page 4, lines 52-54, and page 5, lines 3-5: What really were the exclusion criteria? To be clear, exclusion criteria cannot be the opposite of inclusion criteria. Exclusions are participants who met the inclusion criteria but have other characteristics that confound the outcome. For instance, temporary residents and parents without under-5 children cannot be exclusion criteria. Please, modify this

	section appropriately for clarity. Furthermore, it would also be informative to state why only permanent residents were included in this study.<br>7. Page 5, line 18: What were the reliability and validity measures of the study questionnaire?<br>Results<br>8. Page 8, Lines 48-54: The authors appear to be discussing their findings here instead of just declaring the result as is traditional.<br><br>Aminu Kano Teaching Hospital, Department of Family Medicine<br><br>26-Apr-2025

VERSION 1 – AUTHOR RESPONSE

Reviewer: 1

Dr. Deb Pandey, Agriculture and Forestry University

Comments to the Author

bmjpo-2025-003543_Proof_hi

General

Authors have nicely presented about their attitudes, knowledge, and behaviours of mothers of 5-year-old children towards snakes and snakebite in two rural districts of Sri Lanka (one of the snakebite hotspots in South Asia). However, they need to revise evaluation of household income and data analysis, etc. in method section and first paragraph of the discussion section prior to its accepting for publication. I provided some comments specifically below.

Thank you, we revised the manuscript to address this comment and please see below for our responses for specific comments.

Authors have presented background of this study nicely. But, authors need to define the constraint of this study at the last of discussion that would help to refine the similar studies in future.

We revised the discussion section as per reviewer’s advice. Please see the last paragraph in Discussion section.

Specific

Line 54, 33-34: Please, replace "Ampara and Polonnaruwa districts" with Ampara and Polonnaruwa Districts. It is because common names are capitalized.

Thank you very much!! Done and we have corrected this in other places as well.

Please, place US Dollar equivalent amount in the parenthesis of 200,000 LKR.

Thank you, we have now given the amounts also in USD.

Next, please, elaborate the monthly household income for farmers who receive cash from majority of crops bi-, tri- or four monthly or so on. The rural areas of this study sites would be dominated by farmers. How did you measured or defined monthly income? Please, elaborate this section, herein.

Thank you very much for this comment, we completely agree with the reviewer’s comment. However, for the purpose of the study, we collected data regarding monthly income in families based on what the participants reported as their average monthly household income. Unfortunately, we have not collected a detailed breakdown of the household incomes. To clarify this, we added the following sentence in methods section - “Monthly household income was considered as the total income of all earners in the family during a month, as reported by the participant.” (see in Methods, paragraph 4, lines 2,3)

Page 6, line 3: means

The mean is improper descriptive statistics if the data were non-normally distributed. So, please, state about the normality of your dataset for each of these variables being analyzed and use either mean or median according to the results of normality tests. Otherwise, the method is relatively weak. To improve the method section and finding robust findings, too, I would like to suggest author to apply regression analyses to find and declare the most strong factors affecting attitudes and practices of the parents.

Thank you for the comment; the normality of continuous variables was assessed using the Shapiro-Wilk test and visual inspection of histograms and Q-Q plots. Parental age was found to be normally distributed (Shapiro-Wilk $p = 0.124$, mean 27.0 years (SD = 6.4). Monthly household income, however, was non-normally distributed (Shapiro-Wilk $p < 0.001$, median 265 USD, IQR: 225 USD – 295 USD). The data analysis section was updated appropriately.

Findings of the logistic regression was presented after Table 2. “Logistic regression analysis identified that lack of prior training in first aid (AOR=2.18, 95% CI: 1.12–4.26, p=0.022) and being a mother (AOR=3.07, 95% CI: 1.45–6.51, p=0.003) were independent predictors of extreme fear of snakes. Similarly, lower household income (AOR=2.59, 95% CI: 1.32–5.08, p=0.006) and lower parental education level (AOR=2.17, 95% CI: 1.11–4.24, p=0.023) were independent predictors of reliance on traditional healing practices.”

Page 10, 13-20: The first paragraph of the discussion is to highlight your core findings and support or argue with other similar findings from other rural areas of Sri Lanka or other South Asian countries where socio-economics and cultural practices are similar.

Please, remove "Snakebites in rural Sri Lanka, particularly in districts like Ampara and Polonnaruwa, continue to be a significant public health issue8. These regions, identified as snakebite hotspots due to their vast agricultural lands and favorable habitats for snake populations, witness a high incidence of envenomations annually2. Among the most vulnerable groups are children, whose curiosity and mobility increase their risk of encountering snakes9." From the first section of this paragraph.

Thank you very much, we have now re-written the first paragraph of Discussion section following the suggestion by the reviewer. (See paragraph 1 in Discussion, highlighted lines 1-6)

Reviewer: 2

Dr. Godpower Michael, Aminu Kano Teaching Hospital

Comments to the Author

GENERAL COMMENTS

Snakebite remains a neglected tropical disease. The topic of this study, “Preventive practices and parental attitudes towards snakebite in children in snakebite hotspots of rural Sri Lanka,” is an important contribution to the Sri Lankan literature. The grammatical style of the authors is easy to understand, and the technical presentation of the tables is commendable. The discussion and conclusions are okay. However, there are a few issues in the Introduction, methods, and results sections that need to be addressed.

SPECIFIC COMMENTS

Abstract

1. The aim of the study was not clearly stated.

The aim of the study is now added before Methods section in the abstract “This study aimed to assess parental attitudes, knowledge sources, and preventive practices related to snakebite prevention and management in children living in snakebite-endemic rural regions of Sri Lanka.”

Introduction

2. Here, I struggled to find current evidence on preventive practices and parental attitudes towards snakebite in children. Using the funnel approach, the reader would be interested in seeing a concise description of what is known of this topic at the global, regional, national, and local levels. This helps expose the gap in the current knowledge, which this study is designed to address.

Thank you very much for the comment!! The introduction section was re-written and some of the less appropriate references were replaced with more appropriate references. The limited nature of literature on this aspect of snakebites was also highlighted.

Methods

3. Lines 29-33: It appears that the aim of the study is inconsistent with the one stated in lines 19-25. Please, the aim of the study should be consistently stated for clarity.

Thank you. The aim of the study is now clearly stated both in the abstract and just before the methods section.

4. When was this study carried out?

Data were collected for six months (from October 2024 to March 2025). This is now mentioned under data collection sub-heading in Methods section

5. Page 4, Line 47: Do you mean that p=43 % was obtained from the paper written by Silva et al? If so, which variable does this represent (parental attitude or preventive practices)? Transparency is required in calculating the sample size.

Thank you very much!! We apologise for the typo error; we considered this study which reported preventive practices among Sri Lankan farmers for calculation of the sample size for our study. In order to achieve the most appropriate sample size adjusted for all variables, we considered, the variable (under prevention practices) – storing firewood outside houses that was reported as 75.8% (lowest proportion among all reported variables). We took this proportion as 0.76. The relevant sentence was rewritten as “With a 95% confidence level (Z=1.96), an estimated proportion (P=0.76),

and a 5% margin of error (d=0.05), the sample size was calculated to be 281 participants.”. Thank you once again for pointing this out.

6. In page 4, lines 52-54, and page 5, lines 3-5: What really were the exclusion criteria? To be clear, exclusion criteria cannot be the opposite of inclusion criteria. Exclusions are participants who met the inclusion criteria but have other characteristics that confound the outcome. For instance, temporary residents and parents without under-5 children cannot be exclusion criteria. Please, modify this section appropriately for clarity. Furthermore, it would also be informative to state why only permanent residents were included in this study.

Thank you. This section was revised taking the reviewer’s comment into consideration – “Exclusion criteria included parents who reported having significant cognitive impairments or mental health conditions that might interfere with their ability to accurately recall and report knowledge and practices related to snakebite prevention and management. Parents who were unable to provide informed consent were also excluded. Only permanent residents of the selected districts were included in the study to ensure participants had consistent access to local healthcare services and public health initiatives. Temporary residents, who might not have had the same exposure to the local healthcare system, health programs, or community-specific risk factors, were excluded to maintain the validity of the data.”

7. Page 5, line 18: What were the reliability and validity measures of the study questionnaire?

Thank you, we did not use statistical techniques for this purpose. “The reliability of the questionnaire was ensured through a pre-test with 10 parents from non-study areas to identify any issues with clarity and consistency. Validity was established through expert review for content validity and pre-testing with participants for face validity. Feedback from these processes was used to refine the questionnaire for clarity and relevance.” This information was added under data collection subheading.

Results

8. Page 8, Lines 48-54: The authors appear to be discussing their findings here instead of just declaring the result as is traditional.

Thank you very much, we revised this section as advised and removed the explanation from Results section.

VERSION 2 - REVIEW

**REVIEWER NAME**	***Deb Pandey***
**REVIEWER AFFILIATION**	Agriculture and Forestry University, Department of Veterinary Microbiology and Parasitology
**REVIEWER CONFLICT OF INTEREST**	N/A
**AI DISCLOSURE**	No
**DATE REVIEW RETURNED**	13-May-2025

**GENERAL COMMENTS**	bmjpo-2025-003543.R1_Proof_hi<br>General<br>Authors have adequately revised this manuscript and presented attitudes, knowledge, and behaviors of mothers of 5-year-old children towards snakes and snakebite in two rural districts of Sri Lanka (which is one of the snakebite hotspots in South Asia) with distinct constraints paving ways to improve future similar studies. However, they need to revise the discussion slightly prior to its

	accepting for publication. I provided some comments specifically below.<br><br>Specific<br>Line39-41: Please, replace "Ampara and Polonnaruwa districts" with Ampara and Polonnaruwa Districts. It is because common names are capitalized.<br>see: A descriptive cross-sectional study was conducted in the Ampara and Polonnaruwa districts of Sri Lanka, both of which are rural areas with a high incidence of ...<br><br>Page 12, 36-40: The following section of the discussion can further be improved.<br>"... Culturally sensitive educational interventions and community awareness programs have been shown to reduce fear and improve appropriate responses to snake encounters, ultimately aiding in better snakebite prevention and management10.".<br>The 10th reference is Indian experience. You can extend the coverage of your article's influence to Nepalese population as well where an educational intervention provided a citable contribution in prehospital care, etc. The full pdf is available in: http://103.69.126.140:8080/bitstream/20.500.14356/2003/1/214-Article%20Text-212-1-10-20130822.pdf<br><br>Page 13: Line 9-20: "... Similarly, studies in other resource limited countries, have reported the persistence of traditional beliefs influencing snakebite treatment, including reliance on traditional healers instead of seeking medical attention4,15.. "<br>Please, improve this section as:<br>"... Similarly, studies in other resource limited countries in South Asia and Africa, have reported the persistence of traditional beliefs influencing snakebite treatment, including reliance on traditional healers instead of seeking medical attention4,15. "<br>The recent study of Attitudes, knowledge and practices of traditional snakebite healers in Nepal can be the most suitable source to cite in this section as it is implacable for prevention and control of snakebite in all South Asian and African nations where faith healers influence snakebite management (find its pdf link herein, please: https://doi.org/10.1093/trstmh/trac104).<br><br><br>Page 13: Line 25-30: "..These findings are consistent with studies from other snakebite-endemic regions, such as rural India and Brazil, where access to healthcare is often limited due to long distances, poor infrastructure, and financial constraints5,16.".<br><br>Please, improve this section as:<br>"..These findings are consistent with studies from other snakebite-endemic regions, such as rural India, Nepal, and Brazil, where access to healthcare is often limited due to long distances, poor infrastructure, and financial constraints5,16.".<br><br>for: long distances and d inadequate access to snakebite treatment centres as barrier in Nepalese snakebite management is well documented in: https://doi.org/10.1371/journal.pntd.0011572; https://doi.org/10.1093/trstmh/trac104<br><br>for: financial constraints i.e, the poor financial situation of most families inhabiting snakebite prone areas in Nepal in:

	https://doi.org/10.1093/trstmh/trac104;<br>https://doi.org/10.4269/ajtmh.24-0399

**REVIEWER NAME**	***Godpower Michael***
**REVIEWER AFFILIATION**	Aminu Kano Teaching Hospital, Department of Family Medicine
**REVIEWER CONFLICT OF INTEREST**	N/A
**AI DISCLOSURE**	No
**DATE REVIEW RETURNED**	01-May-2025

**GENERAL COMMENTS**	None

VERSION 2 – AUTHOR RESPONSE

Reviewer: 1

Dr. Deb Pandey, Agriculture and Forestry University

Specific

Line39-41: Please, replace "Ampara and Polonnaruwa districts" with Ampara and Polonnaruwa Districts. It is because common names are capitalized.

Thank you, we revised the manuscript as advised by the reviewer. Please see the highlighted section under the sub-section 'Study design and population' in 'Methods' section.

Page 12, 36-40: The following section of the discussion can further be improved.

"... Culturally sensitive educational interventions and

community awareness programs have been shown to reduce fear and improve appropriate responses to snake encounters, ultimately aiding in better snakebite prevention and management10.".

The 10th reference is Indian experience. You can extend the coverage of your article's influence to Nepalese population as well where an educational intervention provided a citable contribution in prehospital care, etc. The full pdf is available in:

http://103.69.126.140:8080/bitstream/20.500.14356/2003/1/214-Article%20Text-212-1-10-20130822.pdf

Thank you very much for the suggestion. We have now added the following reference (See Ref. 11)

Hamal PK, Pandey DP, Thapa CL. Impact of first aid training in management of snake bite victims in Madi Valley. J Nepal Health Res Counc. 2010. https://doi.org/10.33314/jnhrc.v0i0.214

Page 13: Line 9-20: "... Similarly, studies in other resource limited countries, have reported the persistence of traditional beliefs influencing snakebite treatment, including reliance on traditional healers instead of seeking medical attention4,15.. "

Please, improve this section as:

"... Similarly, studies in other resource limited countries in South Asia and Africa, have reported the persistence of traditional beliefs influencing snakebite treatment, including reliance on traditional healers instead of seeking medical attention4,15. "

The recent study of Attitudes, knowledge and practices of traditional snakebite healers in Nepal can be the most suitable source to cite in this section as it is implacable for prevention and control of snakebite in all South Asian and African nations where faith healers influence snakebite management (find its pdf link herein, please: https://doi.org/10.1093/trstmh/trac104).

Thank you very much!! We have revised the section as advised and the following reference is now added.

Pandey DP, Subedi G, Sapkota S, Dangol DR, Devkota NR. Attitudes, knowledge and practices of traditional snakebite healers in Nepal: implications for prevention and control of snakebite. Trans R Soc Trop Med Hyg. 2023 Mar;117(3):219–28. https://doi.org/10.1093/trstmh/trac104

Page 13: Line 25-30: "..These findings are consistent with studies from other snakebite-endemic regions, such as rural India and Brazil, where access to healthcare is often limited due to long distances, poor infrastructure, and financial constraints5,16.".

Please, improve this section as:

"..These findings are consistent with studies from other snakebite-endemic regions, such as rural India, Nepal, and Brazil, where access to healthcare is often limited due to long distances, poor infrastructure, and financial constraints5,16.".

for: long distances and d inadequate access to snakebite treatment

centres as barrier in Nepalese snakebite management is well documented in: https://doi.org/10.1371/journal.pntd.0011572; https://doi.org/10.1093/trstmh/trac104

Thank you very much!! We have updated the manuscript as advised. Link to the suggested citation (reference 17) is given in superscript.

for: financial constraints i.e, the poor financial situation of most families inhabiting snakebite prone areas in Nepal in: https://doi.org/10.1093/trstmh/trac104; https://doi.org/10.4269/ajtmh.24-0399

Thank you very much for this comment. We have now included the sentence “A study from Nepal further highlighted that the financial burden of snakebite care can exacerbate access barriers, with significant impacts on household economies and care-seeking behaviour”

We add the reference 19 to support this statement - Pandey DP, Adhikari B, Pandey P, Sapkota K, Bhusal MK, Kandel P, Shrestha DL, Shrestha BR. Cost of Snakebite and Its Impact on Household Economy in Southern Nepal. Am J Trop Med Hyg. 2024 Nov 19;112(2):431-440. doi: 10.4269/ajtmh.24-0399. PMID: 39561391; PMCID: PMC11803655.
